# Tear Film Changes Following Anterior Segment Surgery: A Comprehensive Review of Pathophysiology, Clinical Features, and Recovery Time Course

**DOI:** 10.3390/life16071117

**Published:** 2026-07-05

**Authors:** Rafaella Datseri, Nikolaos Ktistakis, Alena Furdová

**Affiliations:** 1 Department of Ophthalmology, Comenius University in Bratislava, Ružinovská 6 Hospital Ružinov, 7th floor, 821 01 Bratislava, Slovakia; alikafurdova@gmail.com; 2 University General Hospital of Heraklion (PAGNI), Voutes–Stavrakia Intersection, Voutes, P.O. Box 1352, 71110 Heraklion, Crete, Greece; 3 State Ophthalmology Department, General Hospital G. Gennimatas, 154 Mesogeion Avenue, Goudi, 11527 Athens, Greece; nickktist@hotmail.gr

**Keywords:** tear film instability, dry eye disease, anterior segment surgery, ocular surface, cataract surgery, refractive surgery, keratoplasty, postoperative complications, tear film recovery

## Abstract

Tear film instability and dry eye disease (DED) are among the most common postoperative complaints after anterior segment surgery. Cataract surgery, corneal refractive procedures, keratoplasty, glaucoma filtration surgery, and pterygium excision can all disrupt ocular surface homeostasis through mechanisms that include corneal denervation, inflammation, goblet cell loss, and meibomian gland dysfunction. The severity and duration of postoperative dry eye vary substantially according to the surgical procedure performed. This review summarises current evidence on the pathophysiology, clinical manifestations, objective tear film changes, and recovery patterns following major anterior segment interventions. Particular emphasis is placed on standardised, non-invasive assessment methods, including tear breakup time, tear meniscus height, meibography, and validated symptom questionnaires. Procedure-specific recovery trajectories are compared to distinguish transient postoperative tear film instability from persistent chronic dry eye disease. Evidence-based management strategies, including preoperative risk stratification, intraoperative optimisation, and multimodal postoperative therapy, are also reviewed. Understanding these distinct recovery patterns may improve surgical planning, patient counselling, early intervention, visual outcomes, and patient satisfaction.

## 1. Introduction

The ocular surface—comprising the corneal and conjunctival epithelia, meibomian glands, and lacrimal apparatus—constitutes a highly integrated physiological unit essential for maintaining corneal transparency, optimal optical performance, and protection against environmental insult. The precorneal tear film, classically described as a trilaminar structure consisting of lipid, aqueous, and mucin layers, is central to this homeostatic system because it provides lubrication, metabolic support, antimicrobial defence, and a smooth refractive interface. Each layer originates from distinct anatomical and cellular sources: the superficial lipid layer is secreted by the meibomian glands; the intermediate aqueous layer is produced by the main and accessory lacrimal glands; and the innermost mucin layer is derived predominantly from conjunctival goblet cells, together with membrane-associated mucins expressed by the corneal and conjunctival epithelia [1,2].

Beyond mucins, the aqueous phase contains high concentrations of lacrimal gland–derived proteins, such as lysozyme, lactoferrin, lipocalin, and secretory IgA, which collectively exert antimicrobial, antioxidant, and immunomodulatory activities. In parallel, the meibomian glands secrete a complex mixture of polar and non-polar lipids that lower surface tension, stabilise the air-tear interface, and limit evaporative loss [1,3]. This stratified and functionally specialised architecture provides a mechanistic basis for the observation that anterior segment surgery induces procedure-specific, compositionally distinct tear film alterations at biochemical and molecular levels, depending on the tissues and neural pathways affected. These surgery-related modifications in inflammatory mediators, mucins, lipids, and neuropeptides are characterised in detail in the Results section.

Anterior segment interventions—including cataract extraction, corneal refractive procedures, keratoplasty, glaucoma filtration surgery, and conjunctival surgery—can perturb ocular surface integrity through mechanisms such as mechanical trauma, corneal denervation, surgery-induced inflammation, and direct damage to epithelial and adnexal structures.

Postoperative dry eye varies in prevalence and severity according to the type of surgery performed. Following corneal refractive surgery, up to 50% of patients may be affected during the immediate postoperative period, with approximately 20% reporting symptoms beyond six months [4]. Cataract surgery usually induces transient tear film disruption, with symptom resolution within three months under routine postoperative care [5]. Keratoplasty and glaucoma filtration surgery are associated with more prolonged ocular surface alterations. This variability reflects differences in surgical trauma, mechanisms of tissue injury, and capacity for ocular surface recovery.

Current evidence indicates that postoperative dry eye is not a uniform entity, but rather a spectrum of tear film alterations with distinct pathophysiological mechanisms and recovery timelines [6]. Standardised non-invasive assessment platforms, including advanced imaging technologies such as anterior segment optical coherence tomography, corneal confocal microscopy, and interferometry-based lipid layer analysis, now allow objective tear film parameters to be characterised in greater detail during long-term follow-up [6]. This shift toward procedure-specific recovery profiles has important implications for preoperative counselling, intraoperative decision-making, and postoperative management.

This comprehensive literature review summarises current evidence on tear film pathophysiology and recovery after major anterior segment surgical interventions. Findings are categorised by surgical procedure to highlight distinct tear film recovery patterns and procedure-specific predisposing factors. Throughout this review, transient procedure-related tear film instability is distinguished from persistent dry eye disease. Evidence for modifiable surgical and perioperative factors affecting tear film outcomes is discussed, together with evidence-based management approaches.

## 2. Materials and Methods

This study was conducted as a comprehensive narrative literature review evaluating tear film changes and postoperative dry eye disease after major anterior segment surgeries, including cataract surgery, corneal refractive procedures (LASIK, FS-LASIK, SMILE, and PRK), keratoplasty, glaucoma filtration surgery, and pterygium excision. The review aimed to summarise current evidence on the pathophysiology, clinical manifestations, objective tear film alterations, recovery timelines, and management strategies associated with postoperative ocular surface dysfunction. A structured literature search was performed using PubMed/MEDLINE, Scopus, Web of Science, and Google Scholar, focusing on English-language articles published between 2015 and March 2026. Priority was given to prospective studies, longitudinal cohort studies, randomised clinical trials, meta-analyses, systematic reviews, and major review articles. Earlier landmark studies were included when necessary to explain fundamental mechanisms such as corneal denervation and lacrimal functional unit disruption.

Search terms included combinations of “dry eye disease,” “tear film instability,” “tear breakup time,” “tear meniscus height,” “ocular surface disease,” “anterior segment surgery,” “cataract surgery,” “refractive surgery,” “LASIK,” “SMILE,” “PRK,” “keratoplasty,” “glaucoma filtration surgery,” and “pterygium excision.” Studies were included if they reported postoperative tear film evaluation using objective parameters such as tear breakup time (TBUT), non-invasive keratographic breakup time (NIKBUT), Schirmer test, tear meniscus height (TMH), lipid layer thickness, meibography, ocular surface staining, anterior segment optical coherence tomography, corneal confocal microscopy, or validated symptom questionnaires including OSDI and DEQ-5. Studies focused exclusively on posterior segment surgery or lacking objective postoperative tear film evaluation were excluded. Data extraction focused on mechanisms of tear film disruption, symptom severity and duration, recovery patterns, risk factors for persistent dry eye, and evidence-based perioperative management strategies, allowing comparison of procedure-specific tear film recovery trajectories and identification of clinically relevant preventive and therapeutic approaches.

## 3. Results

### 3.1. Pathophysiology of Postoperative Tear Film Disruption

#### 3.1.1. Corneal Denervation and Neurotrophic Mechanisms

The predominant mechanism underlying postoperative tear film disruption in many anterior segment procedures is corneal denervation. Corneal refractive surgery, particularly LASIK with flap creation, transects sub-basal and sub-epithelial corneal nerve fibres, thereby damaging sensory pathways involved in reflex tear secretion [6]. Marked corneal denervation occurs intraoperatively, followed by gradual nerve regeneration that may require 3 to 6 months after LASIK [6] and approximately 3 months after cataract surgery [5]. This denervation disrupts three components of the reflex arc:

1. the corneal-lacrimal gland reflex, reducing aqueous tear production;
2. the corneal-blink reflex, decreasing blink frequency and tear distribution; and
3. the blinking-meibomian gland expression reflex, which alters lipid layer secretion [7].

In addition, corneal nerves continuously release neurotrophic factors that support epithelial cell survival. Loss of this neurotrophic support promotes corneal epithelial fragility and delayed wound healing.

Recent studies have identified ferroptosis—an iron-dependent form of regulated cell death—as a novel mechanism that contributes to tear film dysfunction after corneal nerve injury. Unilateral severing of the corneal nerve triggers bilateral lacrimal gland ferroptosis through the VIP/Hif1a/TfR1 pathway, while VIP supplementation inhibits ferroptosis and restores tear secretion [8]. Circadian rhythm disruption also contributes to postoperative dry eye. Downregulation of the circadian core gene Nr1d2 affects lipid metabolism and mitochondrial function in the lacrimal glands, while activation of the SR9011 receptor restores lacrimal gland function and diminishes dry eye symptoms [9].

#### 3.1.2. Inflammatory Mechanisms

Anterior segment surgical interventions induce acute inflammatory responses that affect the tear film. Mechanically induced injury from corneal incisions and epithelial ablation releases damage-associated molecular patterns (DAMPs), promoting innate immune activation and increased tear osmolarity. Elevated tear osmolarity subsequently stimulates TRPV1 (transient receptor potential cation channel subfamily V member 1) and other osmoreceptors at sensory nerve terminals, contributing to ocular surface pain and inflammation [10].

Acute anterior segment inflammation after refractive and cataract surgery can be clinically characterised by increased anterior chamber flare, which typically peaks on postoperative day 1 and normalises within 1 month [11]. Procedures such as SMILE and FS-LASIK may produce transient blood-aqueous barrier dysfunction; however, a direct correlation between perioperative dry eye severity and anterior chamber flare has not yet been established. Proteomic analysis of postoperative tear samples has demonstrated elevated concentrations of MMP-9, IL-1, IL-8, and other inflammatory markers beyond the acute postoperative phase [12].

#### 3.1.3. Ocular Surface Epithelial and Goblet Cell Disruption

Mechanical trauma during surgery damages the corneal and conjunctival epithelium, leading to reduced goblet cell density and altered mucin expression. Goblet cell loss reflects both direct mechanical injury at the time of surgery and subsequent inflammation-mediated apoptosis. Reduced goblet cell density decreases mucin layer thickness, thereby contributing to tear film instability and increased tear osmolarity [13].

Surgical manipulation, particularly suction during LASIK flap creation or conjunctival manipulation during glaucoma filtration surgery, can directly damage goblet cells. Postoperative anti-inflammatory therapy may also influence goblet cell density; for example, corticosteroids may increase goblet cell density, whereas nonsteroidal anti-inflammatory drugs (NSAIDs) may reduce it. In keratoplasty, donor tissue lacks sensory innervation, and potential immune reactivity may contribute to sustained epithelial irregularity and reduced mucin retention [14].

#### 3.1.4. Meibomian Gland Dysfunction

Intraoperative trauma to the lid margin may contribute to postoperative MGD. In addition to physical injury from surgical instruments or specula, lid margin swelling can reduce meibomian gland expressibility. Changes in the lipid composition of secreted meibum have also been implicated after keratoplasty. Reduced meibomian gland expressibility is evident during the first postoperative week and begins to improve within 6 months [14]. However, decreased meibum scores may still be observed 12 months after transplantation. Factors contributing to postoperative MGD include direct thermal damage from operative microscope illumination, inflammation-induced gland dysfunction, and alterations in normal circadian rhythms of meibum production. The severity of MGD varies according to surgical procedure, and available evidence suggests that it is less pronounced after refractive surgery than after keratoplasty.

### 3.2. Tear Film Changes After Cataract Surgery

#### 3.2.1. Epidemiology and Temporal Pattern

Cataract surgery is one of the most frequently performed ocular procedures worldwide. Postoperative dry eye affects approximately 40–90% of patients, depending on the diagnostic criteria, assessment methodology, and timing of evaluation [15]. In most cases, cataract-related tear film disruption is transient, with spontaneous resolution within approximately 3 months [16]. The temporal profile differs from that observed after corneal refractive surgery, typically showing an early postoperative decline in tear film parameters followed by gradual recovery.

#### 3.2.2. Objective Tear Film Parameters

Tear Breakup Time (TBUT): TBUT decreases significantly within the first postoperative week, remains reduced for up to approximately 3 months, and generally returns to preoperative levels thereafter [17]. Comparisons between cataract extraction techniques show minimal differences in tear film outcomes. Phacoemulsification and manual small-incision cataract surgery (SICS) produce similar changes, with TBUT decreasing from 8.6 s preoperatively to 7.7 s at 1 month postoperatively [16]. Despite theoretical advantages related to incision precision, femtosecond laser-assisted cataract surgery (FLACS) has not demonstrated a clear additional benefit for tear film recovery [5].

Tear Volume and Tear Meniscus Height: Schirmer test values may decrease after cataract surgery, indicating reduced basal tear secretion [17]. By contrast, tear meniscus height shows more variable postoperative patterns, with some studies reporting recovery by 3 months [14]. This discrepancy suggests that tear production and tear distribution may be influenced by distinct mechanisms and that anatomical or eyelid-related factors may independently affect tear meniscus measurements.

Corneal Staining: Positive ocular surface staining with fluorescein or lissamine green is frequently observed after cataract surgery and reflects epithelial disruption and localised corneal drying [17]. Corneal and conjunctival staining typically increases during the first postoperative week and correlates with reduced TBUT and higher OSDI scores [16].

#### 3.2.3. Subjective Symptoms and Impact on Quality of Life

Prospective studies have reported modest postoperative increases in Ocular Surface Disease Index (OSDI) scores, for example, from 12.15 preoperatively to 13.79 at 1 month after surgery [15]. Common symptoms include burning, foreign body sensation, photophobia, and blurred or fluctuating vision. Notably, subjective symptoms may correlate poorly with objective tear film parameters. Some patients remain asymptomatic despite subclinical tear film abnormalities, whereas others report substantial discomfort despite apparently normal tear film measurements. This discordance suggests that neuropathic pain mechanisms and altered corneal sensory function may contribute to postoperative ocular discomfort independently of measurable tear film instability.

#### 3.2.4. Risk Factors and Preoperative Predictors

Female sex and pre-existing dry eye are independent risk factors for more severe postoperative dry eye after cataract surgery [12,18]. Baseline tear film abnormalities, particularly reduced tear meniscus height and shortened TBUT, are predictive of a higher probability of postoperative dry eye. Advanced age, previous ocular surface disease, and systemic inflammatory conditions may further increase risk. Careful preoperative assessment and optimisation of tear film function before cataract surgery may therefore improve postoperative comfort and visual outcomes [18].

#### 3.2.5. Mechanism of Injury

Cataract surgery-related tear film disruption is multifactorial. Clear corneal or scleral incisions may disrupt stromal nerve fibres and reduce corneal sensitivity. Surgical manipulation can induce conjunctival inflammation and goblet cell loss, while eyelid speculum use and intraoperative fluid exposure contribute to mechanical stress. Irrigating solutions and postoperative medications, particularly those containing preservatives, may further irritate the ocular surface [13]. The relatively transient nature of cataract-associated dry eye likely reflects the lower degree of corneal denervation compared with refractive surgery, as corneal nerve regeneration after phacoemulsification generally occurs more rapidly than after LASIK.

### 3.3. Tear Film Changes After Refractive Surgery

#### 3.3.1. Epidemiology Across Refractive Techniques

Dry eye disease represents the most common postoperative complication of corneal refractive surgery, with incidence and severity varying among techniques. A meta-analysis of refractive surgery outcomes identified a significant reduction in tear breakup time (TBUT) after LASIK (standardised mean difference [SMD] = −0.3) and femtosecond lenticule extraction (FLEx; SMD = −1.09), whereas reductions after SMILE (SMD = −0.34) and PRK (SMD = −0.11) were not statistically significant [19]. Tear production decreases significantly after LASIK but shows nonsignificant changes after SMILE, FLEx, and PRK [19].

The overall incidence of postoperative dry eye at approximately 2 months after refractive surgery is 41.4%, with significant differences between procedures [20]. Surface ablation procedures, including PRK and transepithelial PRK, demonstrate an incidence of 45.7%, compared with 39.7% for lamellar procedures such as LASIK and FS-LASIK. This difference is likely attributable to greater stromal inflammation and prolonged epithelial healing after surface ablation [20].

#### 3.3.2. LASIK-Induced Tear Film Disruption

Pathophysiology: LASIK involves the creation of a corneal flap using either a mechanical microkeratome or a femtosecond laser, followed by stromal ablation. Flap creation transects corneal nerves in the central and mid-peripheral cornea, resulting in marked denervation of the ablation zone. Among refractive procedures, LASIK therefore represents one of the most substantial surgical insults to corneal innervation [6].

Tear Film Parameters: TBUT decreases significantly within the first postoperative week after LASIK, remains shortened for approximately 3–6 months, and may not fully recover for up to 12 months [21]. Tear meniscus height decreases by approximately 31% at 1 month after FS-LASIK [22]. Non-invasive keratographic breakup time (NIKBUT) decreases by approximately 40% after FS-LASIK [22], compared with more modest reductions after SMILE [22].

Tear film osmolarity increases after LASIK, and sustained elevation over several months may reflect ongoing inflammation and altered tear film composition. Lipid layer thickness decreases acutely after surgery, although recovery varies according to postoperative anti-inflammatory and lubricating therapy [22].

Corneal Nerve Involvement: FS-LASIK produces greater corneal nerve disruption than SMILE, with significant reductions in nerve fibre density, branch density, fibre length, and fractal dimension [22]. Corneal nerve fibre length correlates with dry eye symptoms, measured using the DEQ-5 questionnaire, particularly after FS-LASIK [22]. This association suggests that the degree of corneal denervation may predict symptom severity.

Temporal Recovery: TBUT typically begins to recover approximately 3 months postoperatively, with gradual improvement over 6–12 months [21]. Nevertheless, approximately 20% of patients undergoing LASIK report persistent dry eye symptoms at 6 months or beyond, and a smaller subset experiences chronic symptoms lasting 1 year or longer [4].

Long-Term Outcomes: In a 12-month comparison of dry eye after SMILE and FS-LASIK, TBUT improved by 3 months in the SMILE group (*p* < 0.001) but remained significantly reduced until 6 months after FS-LASIK [23]. At 12 months, both groups showed similar dry eye severity, suggesting that although SMILE enables earlier tear film recovery, long-term dry eye outcomes may converge between techniques [23].

#### 3.3.3. Photorefractive Keratectomy (PRK) and Surface Ablation Techniques

Mechanism: PRK involves epithelial removal followed by stromal ablation without flap creation. Surface ablation techniques avoid flap-related transection of anterior stromal nerves and may therefore reduce corneal denervation compared with LASIK. However, the ablation zone still removes corneal nerve fibres, and prolonged epithelial healing induces substantial postoperative inflammation.

Tear Film Changes: Meta-analytic evidence indicates nonsignificant reductions in TBUT (SMD = −0.11) and tear production (SMD = −0.07) after PRK [19]. Clinical observations suggest faster recovery of tear film stability after PRK than after LASIK. Schirmer scores normalise by 1 month after PRK (18.5 ± 6.75 mm), compared with lower values after LASIK (15.65 ± 6.41 mm) [24]. Non-invasive tear breakup time is numerically higher at 1 month after PRK (12.55 ± 6.07 s) than after LASIK (9.53 ± 6.97 s), although this difference does not reach statistical significance [24]. Tear meniscus height abnormalities are also less frequent after PRK than after LASIK at 1 month (0% versus 20%) [24].

Risk Factors: Independent risk factors for postoperative dry eye after refractive surgery include sex, age 26–30 years, surface ablation procedures, preoperative tear meniscus height < 0.2 mm, and preoperative TBUT < 10 s [20]. Among these factors, surface ablation procedures are associated with a 1.32-fold greater likelihood of postoperative dry eye compared with lamellar procedures [20].

#### 3.3.4. Small Incision Lenticule Extraction (SMILE)

Procedure and Mechanism: SMILE involves femtosecond laser creation of an intrastromal lenticule that is extracted through a small 2–4 mm incision. This technique preserves most anterior corneal nerves, with denervation limited mainly to the lenticule extraction plane and the region surrounding the incision [21].

Tear Film Recovery: SMILE is associated with superior early postoperative tear film stability compared with LASIK across multiple parameters. Tear meniscus height returns to approximately 95% of the preoperative value by 1 month after SMILE, whereas persistent reduction is observed after FS-LASIK and microkeratome LASIK [21]. NIBUT-1 and NIBUT-a decrease significantly on postoperative day 1 in both SMILE and FS-LASIK groups, but show greater recovery by 3 months after SMILE [11].

Dry Eye Symptoms: OSDI scores increase transiently after both SMILE and FS-LASIK, particularly from postoperative day 1 through the first month [11]. At 1, 3, and 6 months postoperatively, SMILE is associated with significantly lower symptom scores than FS-LASIK [23]. The symptomatic advantage of SMILE may exceed differences in objective tear film parameters, suggesting that reduced corneal denervation improves not only tear film stability but also neuropathic pain-related symptoms.

Corneal Nerve Preservation: Short-term in vivo confocal microscopy analyses demonstrate that both FS-LASIK and SMILE reduce corneal nerve fibre density, branch density, fibre length, and fractal dimension; however, SMILE causes significantly less nerve disruption [22]. Preservation of corneal innervation likely contributes to superior tear secretion, meibomian gland function, and blink reflex maintenance after SMILE [21].

Long-Term Outcomes: Although SMILE demonstrates superior tear film stability at 6 months compared with FS-LASIK, long-term follow-up suggests convergence of outcomes by 12 months [23]. This pattern suggests that although corneal reinnervation occurs more slowly after LASIK, most patients eventually achieve similar tear film recovery regardless of technique.

#### 3.3.5. Inflammatory Markers and Tear Biomarkers

Changes in inflammatory and pain-related biomarkers have been detected in postoperative tears after refractive surgery. Five days after Femto-LASIK, concentrations of MMP-9, IL-1, IL-8, and other inflammatory mediators are increased [12]. Decreased levels of substance P and calcitonin gene-related peptide (CGRP) have also been observed in tears 5 days after surgery, likely reflecting acute corneal denervation. Together, these biomarker changes suggest that both inflammatory and neuropathic mechanisms contribute to postoperative dry eye symptoms after refractive surgery.

Postoperative treatment with cyclosporine A has been shown to suppress IFN-γ and TNF-α expression and preserve goblet cell integrity, with recovery of KRT-7 and MUC5AC expression in conjunctival cells [25].

### 3.4. Tear Film Changes After Keratoplasty

#### 3.4.1. Epidemiology and Severity

Keratoplasty includes penetrating keratoplasty (PK), in which full-thickness donor corneal tissue is transplanted, as well as several lamellar techniques, including anterior lamellar keratoplasty (ALK), deep anterior lamellar keratoplasty (DALK), and endothelial keratoplasty variants. Postoperative dry eye following keratoplasty is among the most persistent ocular surface complications after anterior segment surgery, with tear film abnormalities often remaining detectable for longer periods than after cataract or refractive procedures.

#### 3.4.2. Tear Film Parameters Following Penetrating Keratoplasty

Tear Breakup Time: TBUT shows variable changes after keratoplasty. Some studies have reported significant improvement at 1 week and 12 months postoperatively compared with baseline, despite persistent dry eye symptoms [14]. This apparent discordance suggests that TBUT alone may not fully capture tear film quality after keratoplasty. Improved TBUT may partly reflect reduced corneal sensation after removal of the diseased host cornea and transplantation of denervated donor tissue, potentially masking subjective dry eye symptoms during the early postoperative period.

Tear Meniscus and Tear Production: Tear meniscus height and Schirmer I test values often show no significant change from baseline after penetrating keratoplasty, despite clinical evidence of dry eye [14]. This finding indicates that tear volume alone does not determine dry eye severity after corneal transplantation. Tear film stability, lipid layer quality, epithelial surface regularity, and corneal sensory function are likely more clinically relevant determinants.

Corneal Staining and Epithelial Irregularity: Positive ocular surface staining with fluorescein or lissamine green is frequently observed after keratoplasty [7]. Anterior segment optical coherence tomography may demonstrate persistent epithelial irregularity and corneal surface microtopographic abnormalities after transplantation [7]. These changes contribute to tear film instability and may persist for extended postoperative periods.

#### 3.4.3. Meibomian Gland Dysfunction

Meibomian gland dysfunction is a major contributor to postoperative tear film instability after keratoplasty. Meibomian gland expressibility worsens significantly at 1 week and 6 months postoperatively [14]. Meibum quality scores also decline, and lipid layer interferometry demonstrates reduced lipid layer stability [14]. At 12 months postoperatively, partial recovery of corneal sensation may coincide with persistent or worsening MGD, resulting in increased OSDI scores despite improvement in some objective tear parameters [14]. The mechanisms underlying post-keratoplasty MGD likely include direct thermal exposure from operative microscope illumination, mechanical trauma during graft manipulation, disruption of the corneal–meibomian gland reflex due to denervation of transplanted tissue, and chronic postoperative inflammation.

#### 3.4.4. Corneal Nerve Regeneration and Reinnervation

Corneal nerve regeneration after keratoplasty is slow and often incomplete. Donor corneal tissue is initially denervated, and reinnervation requires several months. During this period, impaired corneal sensation may reduce reflex tear secretion and delay epithelial healing. Partial reinnervation typically occurs within 3–6 months postoperatively, with gradual improvement in corneal sensitivity and tear film parameters [7]. However, complete restoration of normal corneal innervation may not occur even after 12 months, which may explain persistent tear film dysfunction and chronic dry eye symptoms in some patients.

#### 3.4.5. Comparison Between Keratoplasty Types

Different keratoplasty techniques may produce varying degrees of tear film disruption. Lamellar procedures theoretically preserve more host tissue and may therefore induce less ocular surface disturbance than penetrating keratoplasty. However, clinical analyses suggest that lamellar keratoplasty can still produce tear film instability, TBUT abnormalities, and MGD patterns similar to those observed after penetrating procedures [7]. The extent of tear film disruption is influenced by graft size, incision characteristics, surgical trauma, degree of corneal denervation, and postoperative inflammation.

### 3.5. Tear Film Changes Following Glaucoma Filtration Surgery

#### 3.5.1. Epidemiology and Surgical Variations

Glaucoma filtration surgery, most commonly trabeculectomy with or without adjunctive antimetabolites such as mitomycin C or 5-fluorouracil, requires conjunctival dissection and controlled wound healing to establish adequate aqueous drainage. Because the conjunctiva contains a substantial proportion of ocular surface goblet cells and plays an essential role in tear film homeostasis, surgical manipulation and postoperative scarring may substantially affect tear film stability.

#### 3.5.2. Conjunctival Goblet Cell Loss

The most distinctive tear film change after glaucoma filtration surgery is goblet cell disruption. Goblet cells produce the mucin layer, which is essential for tear film stability and ocular surface lubrication. During trabeculectomy, conjunctival manipulation may damage goblet cells through direct mechanical trauma and subsequent inflammation-mediated apoptosis. Adjunctive antimetabolite application, although important for reducing bleb scarring, may further impair goblet cell survival and function. This combined mechanical and chemical injury can result in marked goblet cell loss that persists beyond the early postoperative period.

The consequences of goblet cell loss extend beyond the early postoperative phase. Conjunctival changes and reduced goblet cell density after glaucoma surgery are associated with impaired tear film stability, increased tear osmolarity, and altered tear film composition, including reduced mucin concentration [10].

#### 3.5.3. Tear Film Parameters Following Trabeculectomy

Tear film analysis after glaucoma filtration surgery demonstrates patterns distinct from those observed after other anterior segment procedures. Tear breakup time decreases significantly during the immediate postoperative period and may remain shortened for extended intervals. Schirmer test values may also decrease, reflecting reduced aqueous tear production, altered lacrimal gland function secondary to chronic inflammation, and potential antimetabolite-related toxicity.

Postoperative conjunctival hyperaemia reflects ocular surface inflammation and wound-healing activity. Lipid layer parameters may be relatively preserved compared with procedures that directly affect the cornea or eyelid margin, because the meibomian glands are not directly manipulated during trabeculectomy. Nevertheless, mucin deficiency and altered tear film composition secondary to goblet cell loss may disrupt lipid-aqueous interactions and contribute to persistent tear film instability.

#### 3.5.4. Risk Factors and Prognostic Implications

Greater conjunctival manipulation, higher antimetabolite concentration, longer application duration, and multiple prior glaucoma surgeries increase the risk of postoperative dry eye after trabeculectomy. The extent of conjunctival scarring observed intraoperatively may also predict postoperative ocular surface morbidity. Patients with pre-existing dry eye represent a high-risk subgroup, as additional goblet cell loss and conjunctival scarring may precipitate clinically significant and persistent dry eye symptoms.

### 3.6. Tear Film Changes Following Pterygium Excision

#### 3.6.1. Epidemiology and Surgical Context

Pterygium is a benign fibrovascular proliferation of the conjunctiva that may extend onto the corneal surface and alter ocular surface morphology. Surgical excision remains the definitive treatment, particularly in cases associated with visual axis involvement, progressive growth, irregular astigmatism, chronic irritation, or recurrent inflammation. Depending on the surgical technique used, excision may be combined with conjunctival autografting, amniotic membrane transplantation, or other reconstructive approaches. Although pterygium surgery involves manipulation of the conjunctiva and conjunctival bed, its effects on the tear film differ from those of many other anterior segment procedures because removal of the lesion may also restore ocular surface regularity and improve tear distribution.

#### 3.6.2. Preoperative Tear Film Changes in Pterygium

Patients with primary pterygium frequently demonstrate significant preoperative tear film abnormalities. OSDI score, NIBUT, lid margin abnormality, meiboscore, and lipid layer grading have all been reported to differ significantly between patients with pterygium and healthy controls (*p* < 0.01) [26,27]. By contrast, tear meniscus height and Schirmer I test values do not consistently differ between these groups [26]. Multivariate analyses further indicate that NIBUT, meiboscore, and lipid layer grading correlate significantly with pterygium size and thickness [26,28].

These findings suggest that pterygium-related tear film instability is driven predominantly by abnormal ocular surface mechanics, altered tear distribution, chronic local inflammation, and associated meibomian gland dysfunction, rather than by a primary reduction in aqueous tear production.

#### 3.6.3. Postoperative Tear Film Changes

Tear Breakup Time: Following pterygium excision, non-invasive tear breakup time (NIBUT-average) increases significantly at 1, 3, and 6 months postoperatively compared with preoperative values [26]. This improvement in tear film stability is thought to result primarily from restoration of a more regular corneal contour and removal of the mass effect that previously disrupted uniform tear film distribution. However, improvement may not be evident during the immediate postoperative period. When excision is combined with conjunctival autografting, transient early reduction in tear breakup time and tear film stability may occur during the first days to weeks after surgery. This short-term deterioration likely reflects conjunctival manipulation, graft integration, wound healing, and acute ocular surface inflammation. These early changes generally precede a more sustained and clinically meaningful improvement in tear film stability, which typically becomes apparent within 1 to 3 months postoperatively [29,30].

Meibomian Gland Function: Meiboscore may not differ significantly between preoperative and postoperative assessments [26]; however, separate studies have reported significant reductions in meibomian gland dropout after pterygium excision with conjunctival autografting [29,30]. Lipid layer grading also improves postoperatively [26]. Collectively, these findings suggest that although structural meibomian gland scores may remain relatively stable, meibomian gland secretory function and lipid layer quality can improve after the mechanical distortion caused by pterygium is removed.

OSDI and Symptoms: OSDI scores decrease significantly after surgery compared with preoperative assessments [26]. Lid margin abnormalities also improve postoperatively [26]. Overall, pterygium excision is associated with symptomatic improvement in most patients, although early postoperative discomfort may occur during the wound-healing phase.

Correlation with Pterygium Parameters: At 1 month postoperatively, Schirmer test values and tear meniscus height correlate significantly with pterygium size parameters [26]. However, these correlations diminish by 3 and 6 months after surgery [26]. In contrast, OSDI score, NIBUT, meiboscore, and lipid layer grading remain significantly correlated with pterygium parameters at 1, 3, and 6 months postoperatively [26].

#### 3.6.4. Mechanisms of Tear Film Recovery

In contrast to anterior segment procedures that predominantly impair tear film function through corneal denervation, conjunctival injury, or postoperative inflammation, pterygium excision may improve tear film stability by removing a chronic source of mechanical and inflammatory disruption. The pterygium mass distorts corneal surface topography, interferes with regular tear spreading, and may contribute to meibomian gland dysfunction through chronic ocular surface inflammation. Excision of this pathological tissue and restoration of a more regular ocular surface geometry therefore facilitate recovery of tear film stability [26] (Table 1).

**Table 1:** Comparative summary of tear film changes across major anterior segment procedures, adapted from available literature.

Parameter	Cataract Surgery	LASIK	PRK/SMILE	Keratoplasty	Glaucoma Surgery	Pterygium Surgery
Immediate TBUT Change	↓ Day 1	↓ Day 1	↓ Day 1 (less)	↓ Variable	↓ Significant	Transient early ↓, then ↑ improvement
Peak Dry Eye	Week 1–2	Week 1–4	Week 1–2	Month 1–3	Month 1–3	Month 1
Recovery Timeline	3 months	6–12 months	1–3 months	6–12 months	Prolonged (≥6–12 months; often incomplete)	3–6 months
Corneal Denervation Severity	Mild-Moderate	Severe (LASIK)	Mild (SMILE)	Severe	Minimal (direct)	Minimal
Goblet Cell Loss	Moderate	Moderate	Mild (SMILE)	Moderate	Severe	Minimal
Meibomian Gland Effect	Minimal	Moderate	Mild	Severe	Moderate	Improves
Risk for Chronic DED	Low	Moderate	Low	Moderate-High	Moderate-High	Low

↑ indicates increase, ↓ indicates decrease.

### 3.7. Management Strategies for Postoperative Dry Eye

#### 3.7.1. Preoperative Assessment and Optimisation

Preoperative tear film assessment should be performed before anterior segment surgery, particularly in patients with ocular surface symptoms or known risk factors. Evaluation should include objective measures such as TBUT, Schirmer test, tear meniscus height, lipid layer assessment, and meibography, together with validated symptom questionnaires such as OSDI and DEQ-5. Identification of preoperative dry eye signs or symptoms enables targeted treatment before surgery and may influence procedural planning and postoperative management [14,18].

Treatment options include preservative-free artificial tears, punctal plugs, topical cyclosporine A, oral doxycycline, and omega-3 supplementation in selected moderate-to-severe cases. Optimising the ocular surface before surgery may reduce postoperative symptom severity, improve visual quality, and support a smoother recovery course [18].

#### 3.7.2. Postoperative Anti-Inflammatory and Lubricating Therapy

Topical corticosteroids reduce postoperative inflammation and may help preserve goblet cell density. A tapered course over 4–6 weeks or longer may be considered after procedures associated with substantial ocular surface inflammation, such as LASIK, PRK, or keratoplasty, depending on clinical findings and intraocular pressure status. Cyclosporine A 0.05% suppresses tear film inflammation after refractive surgery and helps maintain goblet cell function [25]. After cataract surgery, treatment with cyclosporine A 0.05% has been associated with increased TBUT and lipid layer thickness compared with carboxymethylcellulose alone [31]. Topical lubricants and tear substitutes provide symptomatic relief and reduce tear hyperosmolarity. Aqueous-based formulations, including hyaluronic acid and carboxymethylcellulose, supplement the aqueous layer, whereas lipid-containing formulations help replenish the lipid layer. Frequent lubrication during the acute postoperative period, followed by gradual tapering as inflammation subsides, remains a standard component of postoperative ocular surface care [6].

#### 3.7.3. Meibomian Gland and Lipid Layer Management

Management of MGD and evaporative dry eye includes conservative measures such as warm compresses and gentle eyelid massage to promote meibum secretion. Topical azithromycin and other antimicrobial agents administered at sub-antimicrobial doses may reduce bacterial lipase activity and limit meibum degradation. Intense pulsed light (IPL) therapy and other thermal-based treatments that improve meibomian gland function have shown promise in postoperative ocular surface management [31].

#### 3.7.4. Punctal Occlusion

Temporary or permanent punctal occlusion increases tear retention time and tear meniscus volume, thereby improving tear film stability in selected patients. Temporary punctal plugs may be used to assess therapeutic response before permanent occlusion is considered. Clinical evidence supports punctal occlusion for symptom relief in patients with persistent postoperative dry eye [6]. However, because punctal occlusion may be associated with complications such as canaliculitis or epiphora, it is generally reserved for patients with moderate-to-severe dry eye that is insufficiently controlled with first-line therapies.

#### 3.7.5. Specialised Therapies for Refractive Surgery

Autologous Serum Eye Drops: Autologous serum eye drops and platelet-rich plasma (PRP) contain growth factors, immunoglobulins, and other biologically active components that support epithelial healing and modulate inflammation. Available studies suggest that these therapies may reduce symptoms and improve tear film stability after refractive surgery, particularly in severe postoperative dry eye that is refractory to conventional treatment [6].

Neurotrophic Factor Supplementation: Increasing evidence suggests that topical nerve growth factor (NGF) and other neurotrophic factors may support corneal nerve regeneration and recovery of corneal sensitivity after refractive surgery [6]. Although cenegermin is approved for neurotrophic keratopathy, its role in accelerating recovery from surgery-induced corneal denervation requires further investigation [6].

#### 3.7.6. Surgical Decision-Making and Technique Modifications

Refractive Surgery Technique Selection: Available evidence suggests that SMILE may be preferable to FS-LASIK in patients with preoperative tear film abnormalities, high myopia (≥5 D), or established dry eye risk factors [21]. PRK may also offer faster tear film recovery than LASIK in selected high-risk patients [24].

Incision Considerations: In cataract surgery, clear corneal incisions appear to produce tear film effects comparable to scleral-based approaches. Temporal incision placement may offer additional advantages by reducing surgically induced astigmatism and potentially preserving corneal innervation compared with superior incisions [13].

Adjunctive Antimetabolite Use: In glaucoma surgery, judicious use of mitomycin C, including shorter application duration, lower concentration, or selective application, may reduce goblet cell toxicity while preserving adequate filtration bleb function.

## 4. Discussion

Postoperative tear film instability after anterior segment surgery is multifactorial and is influenced by surgical trauma, baseline ocular surface status, and postoperative care. A central mechanism is disruption of the lacrimal functional unit through corneal nerve injury. Corneal nerve transection impairs the corneal-lacrimal reflex, reduces tear secretion, decreases blink efficiency and meibomian gland stimulation, and delays epithelial healing by removing neurotrophic support. Accordingly, procedures associated with more extensive corneal denervation, such as LASIK and keratoplasty, tend to produce more severe and prolonged dry eye than cataract surgery or SMILE, which relatively better preserve corneal innervation. Recovery generally parallels corneal nerve regeneration: tear film parameters often normalise within approximately 3 months after cataract surgery but may require 6–12 months or longer after LASIK and keratoplasty. These patterns support corneal nerve recovery as a key determinant of tear film restoration. Inflammation represents a second major contributor. Surgical trauma releases inflammatory mediators, increases tear osmolarity, and promotes goblet cell loss and meibomian gland dysfunction, all of which destabilise the tear film. These effects are particularly relevant after surface ablation, glaucoma filtration surgery, and keratoplasty, where conjunctival manipulation and prolonged wound healing may intensify ocular surface damage. In glaucoma surgery, conjunctival goblet cell loss is especially important because it may produce persistent mucin deficiency and long-lasting dry eye after trabeculectomy. Pterygium excision represents a distinct scenario in which tear film parameters may improve because removal of chronic mechanical distortion and restoration of ocular surface regularity may outweigh the transient surgical injury.

Across studies, preoperative ocular surface status consistently predicts postoperative outcomes. Female sex, older age, MGD, pre-existing dry eye, reduced TBUT, and low tear meniscus height are associated with greater symptom burden and slower recovery. These findings support routine preoperative ocular surface assessment and optimisation before anterior segment surgery. Identification and treatment of subclinical dry eye before surgery may improve postoperative comfort and may also enhance refractive accuracy, visual quality, and patient satisfaction. Surgical planning should also account for dry eye risk; for example, SMILE may be preferable to FS-LASIK in high-risk refractive surgery candidates, while careful antimetabolite selection and dosing in glaucoma surgery may reduce long-term conjunctival toxicity.

A frequent discrepancy exists between subjective symptoms and objective tear film measurements. Some patients report substantial discomfort despite apparently normal clinical tests, whereas others remain asymptomatic despite measurable tear film abnormalities. This discordance suggests that neuropathic pain and altered corneal sensory function may contribute independently of conventional tear deficiency. These observations highlight the limitations of single diagnostic metrics and support combined assessment using symptom questionnaires, TBUT, tear meniscus height, meibography, ocular surface staining, and non-invasive imaging. Current evidence remains limited by heterogeneity in study design, follow-up duration, diagnostic criteria, and assessment methods, which complicates direct comparison between surgical procedures. Prospective multicentre studies using standardised diagnostic platforms and uniform dry eye criteria are needed to define reliable recovery benchmarks. Emerging mechanisms, including ferroptosis-related lacrimal gland dysfunction, circadian rhythm disruption, and neurotrophic factor pathways, may provide future therapeutic targets. Clarifying these mechanisms could enable predictive biomarkers and personalised perioperative strategies to prevent chronic postoperative dry eye and improve long-term surgical outcomes.

## 5. Conclusions

Postoperative tear film perturbation encompasses a spectrum of procedure-specific alterations, each characterised by distinct pathophysiological mechanisms and recovery kinetics. Contemporary evidence from standardised diagnostic platforms indicates that dry eye after anterior segment surgery is not a single uniform complication but rather a constellation of overlapping entities that reflect the type, magnitude, and anatomical location of surgical injury. Recognition of these procedure-specific trajectories enables more accurate preoperative risk stratification, improves patient counselling regarding prognosis and symptom duration, and facilitates timely initiation of targeted interventions. Systematic preoperative detection and optimisation of ocular surface disease, together with careful modulation of modifiable intraoperative and postoperative factors, may reduce the incidence of chronic postoperative dry eye and improve patient-reported outcomes. Future prospective standardised studies are needed to define the cellular and molecular mechanisms underlying tear film recovery, identify robust predictive biomarkers of postoperative dry eye, and evaluate emerging therapeutic approaches, including neurotrophic and chronotherapeutic strategies.

## Data Availability

No new data were created or analysed in this study. Data sharing is not applicable to this article.

## Abbreviations

The following abbreviations are used in this manuscript:

DED	Dry eye disease
TBUT	Tear breakup time
NIKBUT	Non-invasive keratographic breakup time
OSDI	Ocular Surface Disease Index
TMH	Tear meniscus height
PRK	Photorefractive keratectomy
LASIK	Laser-assisted in situ keratomileusis
FS-LASIK	Femtosecond laser-assisted in situ keratomileusis
SMILE	Small-incision lenticule extraction
FLACS	Femtosecond laser-assisted cataract surgery
MGD	Meibomian gland dysfunction
IPL	Intense pulsed light
CsA	Cyclosporine A
